# Prognostic significance of trophoblastic differentiation and β-hCG secretion in somatic malignancies of uterine corpus: A systematic review with survival analysis

**DOI:** 10.18632/oncoscience.625

**Published:** 2025-09-04

**Authors:** Mishu Mangla, Seetu Palo, Harpreet Kaur, Poojitha Kalyani Kanikaram, Emine A. Rahiman

**Affiliations:** ^1^Department of Obstetrics and Gynaecology, All India Institute of Medical Sciences, Bibinagar, Hyderabad, India; ^2^Department of Pathology and Laboratory Medicine, All India Institute of Medical Sciences, Bibinagar, Telangana, India; ^3^Department of Obstetrics and Gynaecology, All India Institute of Medical Sciences, Bilaspur, Himachal Pradesh, India; ^4^Division of Pediatric Hematology and Oncology, Kasturba Medical College, Manipal Academy of Higher Education, Manipal, Karnataka, India

**Keywords:** choriocarcinomatous differentiation, endometrial carcinoma, human chorionic gonadotropin, leiomyosarcoma, prognosis, trophoblastic differentiation

## Abstract

Background: Trophoblastic differentiation or beta-human chorionic gonadotropin (β-hCG) secretion in endometrial carcinoma has been associated with poorly differentiated and aggressive tumors; however, the evidence is largely inconclusive. The review aimed to explore the prognostic role of trophoblastic differentiation and β-hCG in non-trophoblastic, primary uterine corpus cancers.

Methodology: A comprehensive electronic search across databases was conducted for all cases of cancers of the uterine corpus that were either associated with elevated levels of β-hCG or showed evidence of trophoblastic differentiation upon microscopy or both. Cases of gestational choriocarcinoma, trophoblastic tumors, tumors other than uterine corpus, and those tumors of uterine corpus but not reporting β-hCG as a marker were excluded. Data regarding patients’ clinic-demographic details, tumor characteristics, β-hCG levels at the time of presentation, and how these values change with treatment, its peak levels, the extent of loco-regional metastasis, details of treatment received, and case fatality were extracted and analysed statistically.

Results: A total of 35 case reports/case series with a total of 40 cases were included in the present review. The mean age at presentation was 57.75 ± 17.22 years. Nulliparity, obesity, hypertension, and diabetes were important risk factors. Post-menopausal or abnormal uterine bleeding was the commonest presenting complaint. Trophoblastic differentiation or β-hCG expression was found to be associated with high tumor grade, poor differentiation, and poor overall patient survival. The lung was the most common site of metastasis.

Conclusions: Trophoblastic differentiation or elaboration of β-hCG in cancers of the uterine corpus is a rarity, and the majority of them are associated with a grim prognosis, secondary to being associated with poor differentiation, early hematogenous dissemination, and resistance to chemo-radiotherapy. Measurement of pre-operative β-hCG may be considered in all cases of endometrial carcinomas and sarcomas, and if found elevated, immunohistochemical examination with β-hCG should be carried out.

## INTRODUCTION

Human chorionic gonadotropin (β-hCG), a glycoprotein hormone, is primarily synthesized by trophoblasts of the placenta. Elevated levels of β-hCG are notably associated with gestational trophoblastic neoplasms and germ cell tumors with trophoblastic elements; that is presence of trophoblastic elements in the tumor shows a strong correlation with elevated serum β-hCG [1]. Apart from its role as a tumor marker, few investigators have correlated its existence to be associated with aggressive tumor biology; however, the evidence is largely inconclusive [1–3]. Also, while trophoblastic differentiation is an established feature in gestational trophoblastic diseases, its occurrence in non-gestational malignancies has garnered increasing attention due to its potential prognostic implications. Despite advancements in understanding the molecular and histopathological features of uterine corpus malignancies, the prognostic significance of trophoblastic differentiation and β-hCG expression remains unclear.

With this background, this systematic review aims to: (i) identify the baseline characteristics of women presenting with β-hCG -secreting somatic uterine malignancies; (ii) critically analyze the role of β-hCG as a biomarker for disease progression (whether β-hCG expression has any correlation to the tumor grade and extent of loco-regional metastasis) and prognosis of such cases (patient outcome post-surgery); (iii) analyzing overall survival rates; and (iv) review the treatment options, which have been successful or unsuccessful in managing such cases, to guide physicians, encountering such rare cases.

## METHODOLOGY

This systematic review was conducted according to the Preferred Reporting Items for Systematic Reviews and meta-analysis (PRISMA) guidelines, utilizing Covidence software (Supplementary Table 1). A comprehensive electronic search of PubMed, Embase, Scopus, Google Scholar, Cochrane database, and Web of Science was conducted for all cases of β-hCG-secreting somatic uterine malignancies reported in the literature from inception till January 2024. A combination search of subject terms was applied, which included “hcg” OR “human chorionic gonadotropin” OR “BHCG” OR “β-hCG” OR “choriocarcinomatous differentiation” OR “trophoblastic differentiation” “choriocarcinoma” OR “syncytiotrophoblast” AND “endometrial cancer” OR “endometrial carcinoma” OR “endometrial adenocarcinoma” OR “uterine cancer” OR “uterine adenocarcinoma” OR “uterine carcinoma” OR “uterine sarcoma” OR “leiomyosarcoma”. The detailed search strategy is provided as Supplementary Table 2. The reference lists of highly cited papers and the citations to these papers were also snowballed to find additional papers on the topic of interest. Two independent primary reviewers (MM, SP) conducted a literature search using predefined eligibility criteria. Given the rarity of trophoblastic differentiation and β-hCG secretion in uterine malignancies, the existing literature is primarily composed of case reports and small case series. These were included in the review due to the limited availability of larger cohort studies or randomized data. Although such studies inherently carry a higher risk of bias, including publication bias and selective reporting, we considered them valuable for identifying histopathological patterns, clinical presentations, and preliminary prognostic trends. No formal risk of bias tool was applied, as standard tools are not suitable for non-comparative case reports; however, we critically appraised each study for clarity in reporting, diagnostic confirmation, and outcome description. We included the peer-reviewed case reports, case series, correspondence, letters to the editor, pre-print reports describing somatic, that is, non-germ cell, endometrial and myometrial malignancies of the uterine corpus which were either associated with elevated levels of β-hCG or showed evidence of trophoblastic differentiation upon microscopy or both. Cases of gestational choriocarcinoma, trophoblastic tumour, tumors other than uterine corpus, and those tumors of uterine corpus not reporting β-hCG as a marker were excluded. While trophoblastic differentiation has been reported in bladder, lung, and ovarian cancers, the focus of this study was limited to uterine corpus malignancies alone due to their distinct hormonal and pathological microenvironment, which may influence trophoblastic differentiation and β-hCG secretion differently compared to other malignancies.

**Figure 1:**
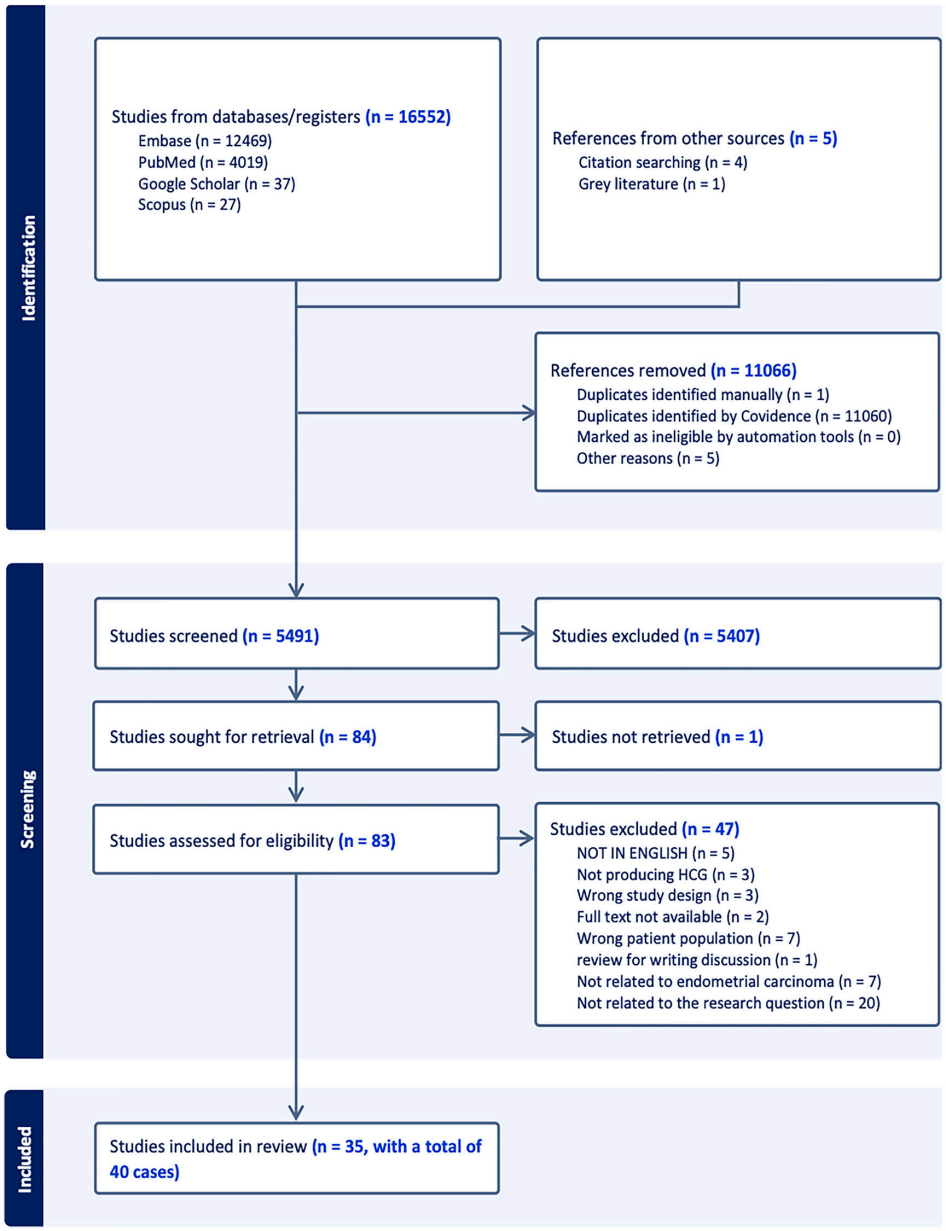
PRISMA Flowchart of the screening process for the cases included in the present review.

In case of any discrepancy, the opinion of the third reviewer (HK) was sought to resolve the issue. Figure 1 represents the PRISMA flowchart of the screening process. The data relating to the patient demographics, clinical presentation, comorbidities associated, serum β-hCG levels, stage and grade of tumour at the time of presentation, immunohistochemical characteristics, the extent of loco-regional metastasis, details of treatment received and how successful it was, clinical progression, and case fatality was extracted and analysed statistically. The data was extracted independently by three reviewers (MM, SP, HK) and cross-verified by a fourth reviewer (PK).

### Statistical analysis

Descriptive statistics were used to summarize patient demographics, tumor characteristics, and biomarker expression (β-hCG positivity, presence of trophoblastic differentiation). When available, survival data such as overall survival (OS), progression-free survival (PFS), or disease-free survival (DFS) were extracted directly from the included studies. Kaplan–Meier survival analysis was performed using IBM SPSS Statistics for Windows, Version 28.0 (IBM Corp., Armonk, NY, USA). Time-to-event data, including overall survival (OS) and progression-free survival (PFS), were calculated from the date of diagnosis to the event (death, recurrence) or last follow-up. Median survival and 95% confidence intervals (CIs) were estimated. Log-rank tests were used for stratified comparisons based on clinicopathological variables, including disease stage, histological subtype, and presence of trophoblastic differentiation or β-hCG expression. Cases with missing survival times or incomplete staging information were excluded from the respective analyses. No imputation was applied.

## RESULTS

Initial database search identified 16552 articles, and after duplicate removal and screening, 35 case reports/case series with a total of 40 cases were included, with clinic-pathological attributes presented in Supplementary Table 3 [4–39].

**Figure 2:**
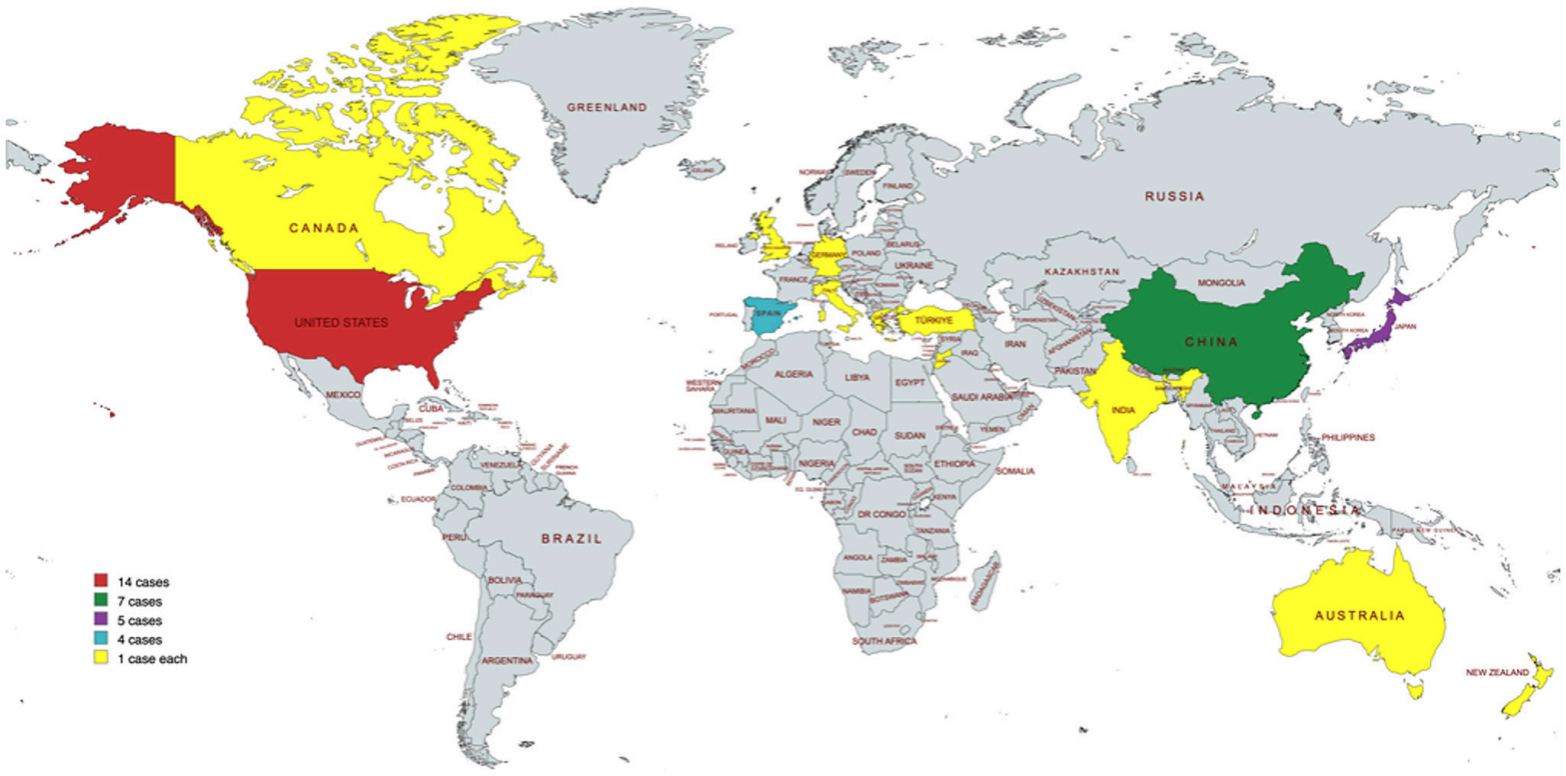
Geographical distribution of the reported cases.

The geographical distribution of the cases is depicted in Figure 2. The mean age at presentation was 57.75 ± 17.22 years, with the median age being 59.5 years (range 24 years to 88 years). Although the obstetric history-related details were missing in 7/40 cases, 21/40 patients were parous, and 12/40 (30%) were nulliparous. Four out of forty patients were obese, with one of them having morbid obesity, with a BMI of 135 kg/m^2^ [12]. Hypertension and diabetes were other important risk factors, present in 10% of the cases. The most common presenting symptom was post-menopausal bleeding (21/40), followed by abnormal uterine bleeding (18/40), which was mostly cyclic heavy menstrual bleeding. There was a notable relationship with hereditary cancers, as none of the patients gave any history of any type of gynaecological or non-gynaecological malignancy in the family. However, one patient had a co-existent breast cancer at the time of diagnosis [22]. In 17/40 cases, β-hCG was not estimated preoperatively, because the authors did not suspect choriocarcinomatous differentiation, and it was detected incidentally on pathology.

Out of the cohort of 40 cases, 34 were of endometrial adenocarcinoma (Endometrioid adenocarcinoma = 21; Serous adenocarcinoma = 5; Clear cell adenocarcinoma = 2; Poorly or undifferentiated adenocarcinoma = 6) exhibiting varying proportions of choriocarcinomatous/trophoblastic differentiation (focal to 85%). Upon immunohistochemistry, the choriocarcinomatous/trophoblastic component was invariably positive for β-hCG and pan-cytokeratin. The adeno-carcinomatous component exhibited positivity for epithelial markers such as pan-cytokeratin (AE1/AE3) and epithelial membrane antigen (EMA). p53 overexpression was found in 10 cases. The remaining 6 cases comprised leiomyosarcoma (*n* = 3), carcinosarcoma (*n* = 2), and an undifferentiated sarcoma (*n* = 1), out of which only two revealed definitive choriocarcinomatous/ trophoblastic differentiation [12, 13]. However, all except one [39] showed focal β-hCG positivity. The most frequent histological subtype of the trophoblastic component was choriocarcinomatous-like, except for a single case, which revealed epithelioid trophoblastic tumor–like areas as well [22].

Approximately two-thirds of the tumors (*n* = 27/40; 67.5%) were of high FIGO grade. Of the 21 endometrioid-type adenocarcinomas, only 5 were of low grade (G1), and 12 cases exhibited higher grade morphology (4 were G2, 8 were G3). Histologic grade was not mentioned in 4 cases. All the cases of uterine carcinosarcoma (*n* = 2), leiomyosarcoma (*n* = 3), and undifferentiated sarcoma (*n* = 1) were of high grade. pT stage was not specified in 19 cases, and the remaining cases presented with varying pT stages (pT1 = 12; pT2 = 1; pT3 = 7; pT4 = 1). The majority of the cases of adenocarcinoma had varying degrees of myometrial invasion (7 cases had less than 50% myometrial involvement; 9 cases had more than 50% myometrial involvement; status of invasion not mentioned in 14 cases), with 3 cases showing 100% involvement with serosal implants. Absence of myometrial invasion was documented in only a single case, but it was pT3a, that is, it showed either serosal implants or adnexal involvement (not specified in the article) [30].

Lung was the most common site of metastasis (37.5%), followed by pelvis (17.5%), and peritoneal cavity, including bowel, or omentum (12.5%). Brain and bone metastasis were found in 7.5% of patients each. Nodal metastasis (retroperitoneal pelvic lymph nodes or supraclavicular lymph node metastasis) was also found in 12.5% of the patients. Twenty-two cases (55%) revealed lympho-vascular space invasion (LVSI) upon microscopy. Although the metastatic site showed exclusive choriocarcinomatous component in the majority of the cases, a few patients showed mixed tumor even at the site of metastasis.

The authors report using varying combinations of surgery, chemotherapy, and radiotherapy in the treatment of cases. While the majority have employed staging surgery followed by post-operative chemotherapy, as the mainstay of treatment (20/40), surgery alone (12/40), surgery and post-operative radiotherapy (5/40), or radiotherapy alone (2/40), were also used by a few authors. Interestingly, in 4/40 cases, neo-adjuvant chemotherapy was used, and 3/4 (75%) of these survived. All three cases where a combination of Etoposide, Methotrexate, Actinomycin-D, Cyclophosphamide and Vincristine (EMACO) was used as neoadjuvant chemotherapy, survived, and one case where Bleomycin, etoposide and platinum (BEP) was used as neoadjuvant chemotherapy, died at 6 months after disease diagnosis due to cerebral metastasis leading to haemorrhage. In one case [6], use of neoadjuvant radiotherapy has been done, although the patient died at 1 year, again due to widespread metastasis. Various chemotherapy options have been utilised, mainly based on treating physicians’ discretion, as there are no standard treatment guidelines. Among the patients who survived, EMACO was the most frequently used post-surgery chemotherapy, followed by various combinations of Docetaxel, Gemcitabine, Nedaplatin, Cisplatin/carboplatin, and Paclitaxel. Radiotherapy has been utilised as adjuvant treatment in some cases, predominantly to control bleeding (haemostatic radiotherapy).

The average time duration to the development of the first recurrence/metastasis was approximately 4.5 months. No recurrence was noted in 11 patients, and 4 were lost to follow-up. 19/40 (47.5%) of the patients died of the disease, at an average of 9.8 months after first diagnosis. The most common cause of death was complications related to metastasis (intracerebral haemorrhage, bowel obstruction, or pulmonary embolism). Two were alive at 6 months but with metastasis-related complications. 12/40 (30%) were alive and healthy, with no complications and with no disease relapse. In these patients, the average disease-free survival was 26.5 months. In 7/40 cases, either the details were not mentioned regarding survival or mortality or the patients were lost to follow-up.

**Figure 3:**
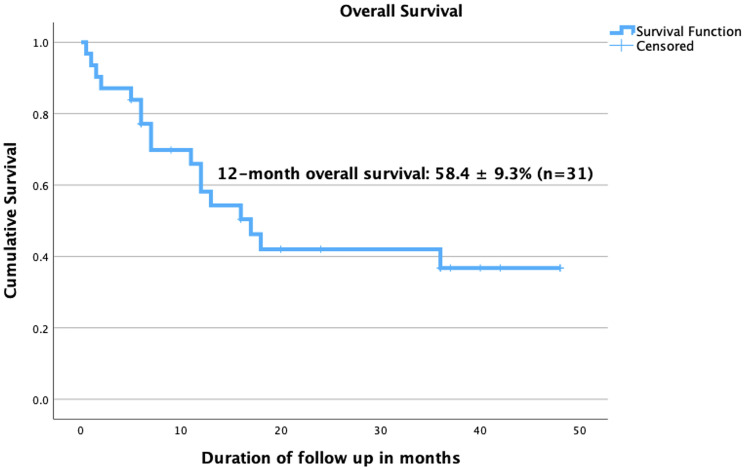
Kaplan-Meier survival analysis, showing the 12-month overall survival analysis for cases of Somatic Malignancies of Uterine Corpus secreting β-hCG. ^*^Number at risk could not be displayed due to inconsistent time-point data across the included cases.

We performed a cumulative survival analysis of 31 cases (with required data points) and found poor survival rates (Figure 3). The mean survival duration in months was 24.7 ± 3.7 (95% Confidence interval: 17.5–31.9) while the median survival duration in months was 17 ± 3.58 (95% Confidence interval: 95% CI 9.97–24.03). The analysis examined multiple prognostic factors in patients, including trophoblastic differentiation, tumor grade, subtype, LVSI, and disease stage, assessing their impact on survival outcomes. Patients with trophoblastic differentiation had a higher 2-year overall survival (OS) rate (41% ± 10.7%) compared to those without (25% ± 20.4%). However, the log-rank test (Mantel-Cox) showed no statistically significant difference (*p* = 0.306). Although the observed survival was higher in low-grade tumors (60% ± 21.9%) compared to high-grade tumors (30.95% ± 11.4%), this difference was not statistically significant (*p* = 0.28). The single case of intermediate-grade tumor was removed from analysis due to its limited representation. For tumor subtype, survival rates varied among different histologic subtypes of the primary tumor. Leiomyosarcoma had the worst prognosis, with all patients in this category, dying. The survival rates for other subtypes ranged from 37.3% ± 13.9% (endometrioid carcinoma) to 50% ± 35.4% (poorly differentiated adenocarcinoma, clear cell adenocarcinoma, uterine carcinosarcoma, and serous carcinoma). However, no significant difference was found (*p* = 0.76). The data regarding LVSI was available for only 21 patients, limiting its interpretability. In our cohort, patients with LVSI had a 2-year OS of 52.8% ± 12.4%. Stage of disease at the time of presentation was the only factor significantly associated with survival (*p* = 0.005). Stage I patients had the best 2-year survival (52.2% ± 16.3%, mean survival 34.4 months), while Stage II and IV had a 0% survival rate. Stage III patients had a 2-year OS of 20.8% ± 18.45%, with a mean survival of 15.5 months. In summary, while trophoblastic differentiation and tumor grade showed trends favouring better survival, only disease stage was significantly associated with survival outcomes.

## DISCUSSION

This systematic review reaffirms that the occurrence of trophoblastic differentiation in endometrial adenocarcinoma is extremely rare (only 34 cases identified), keeping in mind that endometrial carcinoma is the third most common gynecological cancer. The presence of trophoblastic differentiation in carcinosarcoma or sarcoma of the uterine corpus is still rarer (a total of 6 cases published to date). These tumors are found predominantly in the post-menopausal age group, with hypertension, diabetes mellitus, and nulliparity being important risk factors. Post-menopausal bleeding is the commonest presenting symptom. These tumors frequently exhibit poor differentiation, early spread through the bloodstream, resistance to chemotherapy/radiotherapy, and grim overall survival rates.

Amongst the 34 endometrial adenocarcinomas, all exhibited choriocarcinomatous/ trophoblastic components, upon microscopy and immunohistochemical examination, and in 25 of them, increased serum or urinary levels of β-hCG were documented either pre- or post-operatively. Hence, increased levels of β-hCG are a strong marker of choriocarcinomatous/ trophoblastic differentiation and should prompt the pathologist to perform adequate tumor sampling followed by meticulous microscopic search for these areas within the tumor mass. Although trophoblastic differentiation is found more often associated with poorly differentiated adenocarcinoma, it can occur in cases of endometroid (G1/G2/G3), serous, and clear cell adenocarcinoma of the endometrium, as well. If microscopic features are dubious for choriocarcinomatous/ trophoblastic differentiation, immunohistochemistry with β-hCG can be used to document the same. In this context, a few cases, especially sarcomas, might not reveal trophoblastic areas on light microscopy but exhibit immunopositivity for β-hCG [16]. In a recently reported case of uterine leiomyosarcoma with no microscopic evidence of trophoblastic differentiation, there was an increase in serum β-hCG levels post-operatively, for which the authors hypothesized that the β-hCG elevation could be due to disease progression to metastatic disease and biological changes in the metastatic clones encompassing further genetic mutations [39].

The genetic mechanisms underlying choriocarcinomatous differentiation in somatic neoplasms also remain poorly elucidated. Various theories have been proposed, none achieving widespread acceptance. Notably, an analysis has revealed shared genetic alterations between the endometrioid and choriocarcinomatous components within a single tumor, indicating a common clonal origin [2]. Furthermore, the presence of numerous additional genetic alterations in the choriocarcinomatous component suggests significant neoplastic progression from the original endometrioid carcinoma. Other studies have shown that TP53 abnormalities, detected either through immunohistochemistry or molecular analysis, may be present in both components, supporting the notion of a clonal relationship between them [12, 17, 18]. In our review, p53 overexpression was noted in 10 cases. Furthermore, recent molecular evidence indicates that the endometrioid subtype of endometrial adenocarcinoma, known as type 1 tumors, is linked to hyperestrogenism, while serous and clear cell carcinomas of the endometrium, categorized as type 2 tumors, primarily result from mutations in the tumor suppressor gene p53 [17, 18]. We observed two cases of endometrioid adenocarcinoma with trophoblastic differentiation, demonstrated p53 overexpression in both the components, were of higher grade (G3) and stage (pT3a) [21, 30]. In endometrial cancer, overexpression of p53 is a significant prognostic factor [40], and its overexpression in these tumors with trophoblastic differentiation plausibly contributes to dismal prognosis.

Regarding disease progression, we found that the majority of tumors had already undergone distant metastasis at the time of diagnosis itself. The heightened expression of β-hCG fosters the movement and infiltration of cancer cells, encouraging their spread to peritoneal xenografts. Furthermore, increased levels of β-hCG can prompt the transformation of cancer cells from a tightly bound epithelial state, characterized by specific biomarkers, to a more mobile phenotype, marked by mesenchymal indicators, a process known as epithelial-mesenchymal transition [41, 42]. This further highlights the fact that immunohistochemistry for β-hCG may be considered for all cases of endometrial adenocarcinoma with trophoblastic differentiation, as this can be considered an important prognostic factor. Although endometrial adenocarcinoma detected at an early stage is known to have a good prognosis, cases with trophoblastic differentiation present with widespread metastasis, even at an early stage. Consistent with our findings, few investigators, utilizing β-hCG as a biomarker, have also found that somatic carcinomas with trophoblastic differentiation at non-uterine sites also demonstrated a notable correlation with poor differentiation, progressed tumor stage, early hematogenous dissemination, resistance to chemotherapy and radiotherapy, and decreased disease-specific overall survival [2, 3, 43–46]. Of note, it has to be kept in mind that the primary tumor type also plays a role in prognosis. Xie Y et al. reported a case of FIGO grade 1 endometrioid adenocarcinoma with significant choriocarcinomatous differentiation showing no recurrence or metastasis after 3 years post-surgery [37]. This relatively better outcome might be attributed to the low-grade nature of the primary tumor.

While β-hCG expression has been associated with more aggressive tumor behavior in certain cases, its role as a universal prognostic marker remains uncertain due to variability in reported findings. The sensitivity and specificity of β-hCG testing in predicting oncological outcomes have not been consistently established across studies, raising concerns about the potential for false positives, unnecessary additional investigations, patient anxiety, and increased healthcare costs. Hence, β-hCG testing should likely be considered in a case-by-case manner, rather than as a universal screening tool for all endometrial carcinomas. Testing may be more appropriate in cases with histopathological or clinical features suggestive of trophoblastic differentiation or aggressive tumor behavior.

Currently, there are no guidelines that guide the physician regarding which chemotherapy is best for such cases of somatic malignancies with trophoblastic differentiation. Most of the chemotherapy options used by physicians in the above-mentioned cases are predominantly based on research in cases of gestational choriocarcinoma. In compliance with other reported cases of non-gestational choriocarcinoma, the present study also found that these cases were poorly responsive to both chemotherapy and radiotherapy. Although these tumors responded poorly to most of the chemotherapy regimens, 3/4 cases, where neoadjuvant chemotherapy was used before surgery, showed a favourable prognosis. Neoadjuvant chemotherapy with EMACO, followed by staging surgery, and later adjuvant chemotherapy with EMACO or a combination of paclitaxel with platinum-based compounds emerged as the treatment with the most favourable prognosis. Other chemotherapy options included BEP, and varied combinations of 5-fluorouracil, methotrexate, and Adriamycin. The process of dedifferentiation observed in advanced stages of certain malignancies facilitates the emergence of ectopic expression of β-hCG, as outlined by Cole and Butler in 2012 [47]. As tumors undergo greater dedifferentiation, their cell replication tends to become more primitive. This phenomenon potentially elucidates why chemotherapy regimens targeting fundamental cell replication, such as EMACO, can be efficacious [48]. Within the EMACO chemotherapy regimen, actinomycin D serves as a crucial agent, acting as a potent inhibitor of transcription. It is likely a significant component of EMACO responsible for influencing the transcription of β-hCG. Consequently, this process may lead to the elimination of cells within the clear cell component of the patient’s tumor [48, 49]. A recent review by Mukherjee D et al. highlighted the anti-tumor potential of naturally derived compounds in cervical cancer, including their roles in modulating drug resistance, miRNAs, angiogenesis, metastasis, and apoptosis [50]. Exploring similar therapeutic strategies in uterine corpus tumors with trophoblastic differentiation could pave the way for safer and more effective cancer treatments.

**Figure 4:**
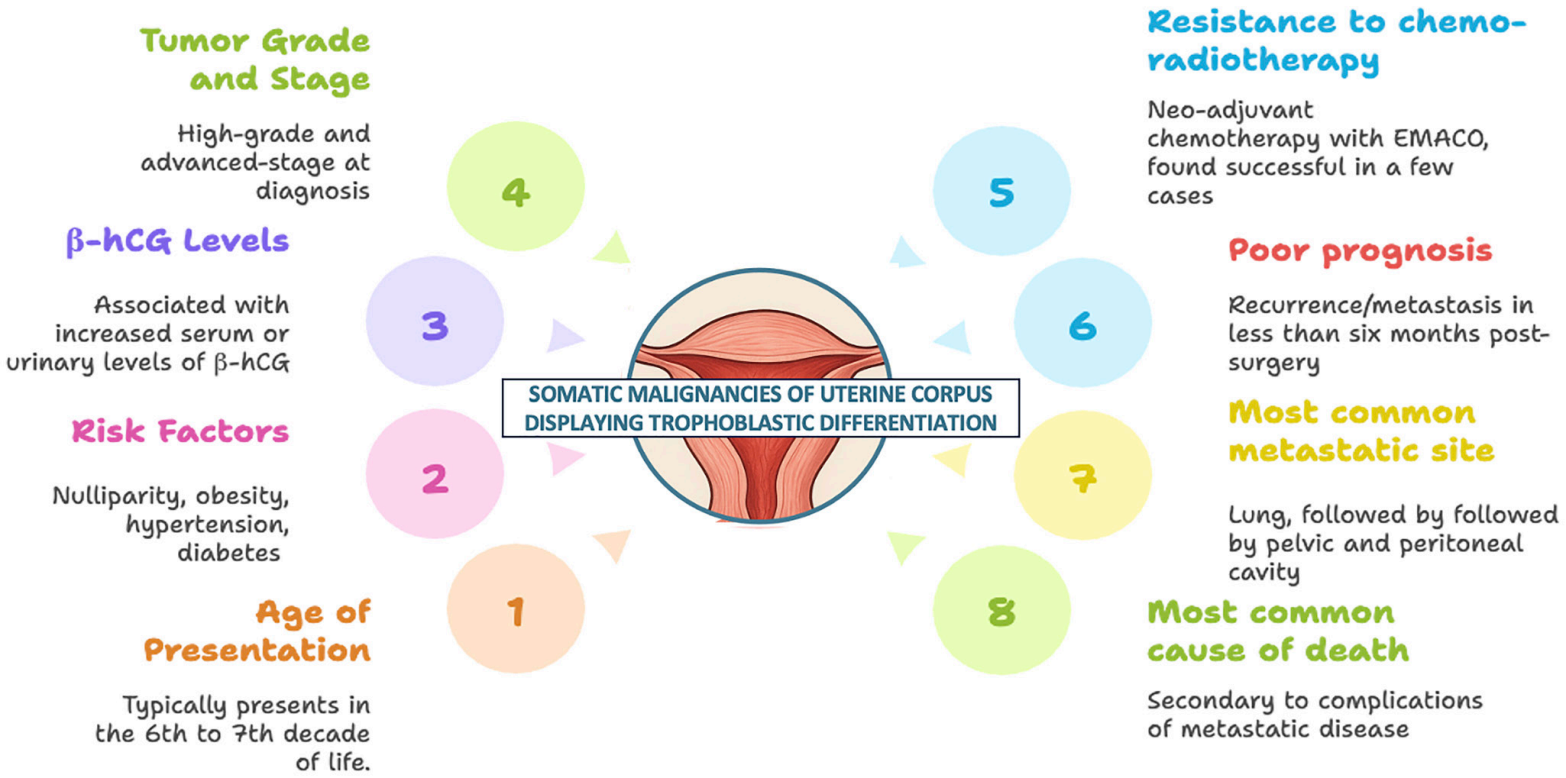
Summary of the key findings of the study.

This study represents the first comprehensive systematic review to compile and analyze all cases of trophoblastic differentiation and also β-hCG secretion (even without trophoblastic differentiation), in somatic tumors of the uterine corpus. Further, we also did survival analysis and found a cumulative 12-month overall survival of less than 60%. The key findings of the study are summarized in Figure 4. The Kaplan–Meier survival analysis adds an exploratory dimension to our review and provides preliminary insights into prognostic patterns in these rare uterine tumors. However, the findings must be interpreted with caution due to the small sample size and resultant wide confidence intervals. We acknowledge that survival comparisons are limited in statistical power and primarily hypothesis-generating. Notably, only disease stage emerged as a significant predictor of survival, underscoring its established prognostic role even in histologically rare variants. The lack of significant association between other variables and survival may reflect the limited number of analysable cases rather than the absence of effect. The review diligently scoured extensive electronic databases to identify all globally published cases available in English, but the data encountered presented a diversity of forms, leading to predominantly descriptive rather than analytical analysis, thereby constraining the depth of the review. The findings of this review must be interpreted in light of the methodological limitations associated with the included literature. The predominance of case reports and small case series, while informative in rare conditions, limits the strength of generalizable conclusions. Such studies are susceptible to bias due to their descriptive nature, lack of control groups, and potential for selective publication of unusual or aggressive cases. Moreover, clinical outcomes and survival data were inconsistently reported. Nevertheless, these reports collectively offer valuable insights into the histological spectrum and possible clinical implications of trophoblastic differentiation and hCG expression in uterine malignancies. Also, heterogeneity across included studies poses a challenge, as variations in study design, patient populations, histological subtypes, and methods of β-hCG detection may influence the consistency of findings. Differences in immunohistochemistry protocols, cut-off values for positivity, and assay sensitivities across studies may affect the comparability of results. Another limitation is the limited availability of survival data in some studies, which restricts the ability to perform comprehensive analyses on disease-free and overall survival. Future multicentre registries or pooled analyses may be necessary to validate these preliminary observations.

## CONCLUSIONS

To conclude, trophoblastic differentiation or elaboration of β-hCG in somatic malignancies of the uterine corpus significantly impacts the prognosis and treatment outcomes of affected individuals. These tumors are often associated with poor differentiation, early hematogenous dissemination, and resistance to chemoradiotherapy. Trophoblastic differentiation and lower tumor grade showed trends favouring better survival; only disease stage was significantly associated with survival outcomes. While EMACO shows promise, its superiority over other regimens remains uncertain due to limited comparative data. Further studies are needed to establish its optimal use across diverse patient profiles. Future studies comparing EMACO with other established protocols, such as EMA-EP or FAEV, are essential to determine the most effective regimen across diverse patient populations. Until such data are available, the current recommendation for EMACO should be interpreted with caution, acknowledging the need for further comparative research. More research is required in cases of somatic carcinomas with trophoblastic differentiation, predominantly in areas of treatment, as presently there are no guidelines to help clinicians decide the best adjuvant chemotherapy or radiotherapy.

## SUPPLEMENTARY MATERIALS




