# Supplementary material for: Prognostic significance of trophoblastic differentiation and β-hCG secretion in somatic malignancies of uterine corpus: A systematic review with survival analysis
Source: Oncoscience. 2025 Sep 4;12:96–106. doi: 10.18632/oncoscience.625 (PMC12410977; doi:10.18632/oncoscience.625)
Supplement: Supplementary file 1 [file oncoscience-12-625-s001.pdf]

Prognostic significance of trophoblastic differentiation and β-hCG secretion in somatic malignancies of uterine corpus: A systematic review with survival analysis

SUPPLEMENTARY MATERIALS

Supplementary Table 1: PRISMA 2020 Checklist for the study

Section and Topic	Item #	Checklist item	Location where item is reported
**TITLE**			
Title	1	Identify the report as a systematic review.	Mentioned in the title
**ABSTRACT**			
Abstract	2	PRISMA 2020 for Abstracts checklist.	Page 3 and 4
**INTRODUCTION**			
Rationale	3	Describe the rationale for the review in the context of existing knowledge.	Page 5
Objectives	4	Provide an explicit statement of the objective(s) or question(s) the review addresses.	Page 5
**METHODS**			
Eligibility criteria	5	Specify the inclusion and exclusion criteria for the review and how studies were grouped for the syntheses.	Page 6
Information sources	6	Specify all databases, registers, websites, organisations, reference lists and other sources searched or consulted to identify studies. Specify the date when each source was last searched or consulted.	Page 5
Search strategy	7	Present the full search strategies for all databases, registers and websites, including any filters and limits used.	Page 5 and 6
Selection process	8	Specify the methods used to decide whether a study met the inclusion criteria of the review, including how many reviewers screened each record and each report retrieved, whether they worked independently, and if applicable, details of automation tools used in the process.	Page 6
Data collection process	9	Specify the methods used to collect data from reports, including how many reviewers collected data from each report, whether they worked independently, any processes for obtaining or confirming data from study investigators, and if applicable, details of automation tools used in the process.	Page 6
Data items	10a	List and define all outcomes for which data were sought. Specify whether all results that were compatible with each outcome domain in each study were sought (e.g. for all measures, time points, analyses), and if not, the methods used to decide which results to collect.	The data relating to the patient demographics, clinical presentation, comorbidities associated, serum β-hCG levels, stage and grade of tumour at the time of presentation, immunohistochemical characteristics, the extent of loco-regional metastasis, details of treatment received and how successful it was, clinical progression and case fatality was extracted. All results that were compatible with each outcome domain in each study were sought
	10b	List and define all other variables for which data were sought (e.g. participant and intervention characteristics, funding sources). Describe any assumptions made about any missing or unclear information.	Geographical area, data of publication

Study risk of bias assessment	11	Specify the methods used to assess risk of bias in the included studies, including details of the tool(s) used, how many reviewers assessed each study and whether they worked independently, and if applicable, details of automation tools used in the process.	Not applicable as meta-analysis was not performed
Effect measures	12	Specify for each outcome the effect measure(s) (e.g. risk ratio, mean difference) used in the synthesis or presentation of results.	Not applicable as meta-analysis was not performed
Synthesis methods	13a	Describe the processes used to decide which studies were eligible for each synthesis (e.g. tabulating the study intervention characteristics and comparing against the planned groups for each synthesis (item #5)).	Not applicable as meta-analysis was not performed
	13b	Describe any methods required to prepare the data for presentation or synthesis, such as handling of missing summary statistics, or data conversions.	Not applicable as meta-analysis was not performed
	13c	Describe any methods used to tabulate or visually display results of individual studies and syntheses.	Data presented as Table 1
	13d	Describe any methods used to synthesize results and provide a rationale for the choice(s). If meta-analysis was performed, describe the model(s), method(s) to identify the presence and extent of statistical heterogeneity, and software package(s) used.	Not applicable as meta-analysis was not performed
	13e	Describe any methods used to explore possible causes of heterogeneity among study results (e.g. subgroup analysis, meta-regression).	Not applicable as meta-analysis was not performed
	13f	Describe any sensitivity analyses conducted to assess robustness of the synthesized results.	Not applicable as meta-analysis was not performed
Reporting bias assessment	14	Describe any methods used to assess risk of bias due to missing results in a synthesis (arising from reporting biases).	Not applicable as meta-analysis was not performed
Certainty assessment	15	Describe any methods used to assess certainty (or confidence) in the body of evidence for an outcome.	Not applicable as meta-analysis was not performed
**RESULTS**			
Study selection	16a	Describe the results of the search and selection process, from the number of records identified in the search to the number of studies included in the review, ideally using a flow diagram.	Presented as figure 1 (PRISMA flowchart)
	16b	Cite studies that might appear to meet the inclusion criteria, but which were excluded, and explain why they were excluded.	No studies meeting the inclusion criteria were excluded
Study characteristics	17	Cite each included study and present its characteristics.	Cited under TABLE 1
Risk of bias in studies	18	Present assessments of risk of bias for each included study.	Not applicable as meta-analysis was not performed
Results of individual studies	19	For all outcomes, present, for each study: (a) summary statistics for each group (where appropriate) and (b) an effect estimate and its precision (e.g. confidence/credible interval), ideally using structured tables or plots.	Data presented as TABLE 1
Results of syntheses	20a	For each synthesis, briefly summarise the characteristics and risk of bias among contributing studies.	Not applicable as meta-analysis was not performed
	20b	Present results of all statistical syntheses conducted. If meta-analysis was done, present for each the summary estimate and its precision (e.g. confidence/credible interval) and measures of statistical heterogeneity. If comparing groups, describe the direction of the effect.	Not applicable as meta-analysis was not performed
	20c	Present results of all investigations of possible causes of heterogeneity among study results.	Not applicable as meta-analysis was not performed
	20d	Present results of all sensitivity analyses conducted to assess the robustness of the synthesized results.	Not applicable as meta-analysis was not performed
Reporting biases	21	Present assessments of risk of bias due to missing results (arising from reporting biases) for each synthesis assessed.	Not applicable as meta-analysis was not performed
Certainty of evidence	22	Present assessments of certainty (or confidence) in the body of evidence for each outcome assessed.	Not applicable as meta-analysis was not performed

**DISCUSSION**			
Discussion	23a	Provide a general interpretation of the results in the context of other evidence.	Page 8 to 11
	23b	Discuss any limitations of the evidence included in the review.	Page 15, 16
	23c	Discuss any limitations of the review processes used.	Page 15, 16
	23d	Discuss implications of the results for practice, policy, and future research.	Page 15, 16
**OTHER INFORMATION**			
Registration and protocol	24a	Provide registration information for the review, including register name and registration number, or state that the review was not registered.	the review was not registered
	24b	Indicate where the review protocol can be accessed, or state that a protocol was not prepared.	the review protocol can be accessed
	24c	Describe and explain any amendments to information provided at registration or in the protocol.	Not applicable
Support	25	Describe sources of financial or non-financial support for the review, and the role of the funders or sponsors in the review.	No financial support
Competing interests	26	Declare any competing interests of review authors.	None declared
Availability of data, code and other materials	27	Report which of the following are publicly available and where they can be found: template data collection forms; data extracted from included studies; data used for all analyses; analytic code; any other materials used in the review.	data extracted from included studies and subsequently used for analysis is presented as TABLE 1

Supplementary Table 2: Search strategy

((“hcg” OR “human chorionic gonadotropin” OR “beta hCG” OR “β-hCG” OR “B-hCG” OR “BHCG” OR “hCG expression” OR “hCG production” OR “chorionic gonadotropin”[MeSH])) OR (“trophoblastic differentiation” OR “trophoblastic elements” OR “trophoblastic features” OR “trophoblastic phenotype” OR “trophoblastic marker*” OR “syncytiotrophoblast” OR “choriocarcinomatous differentiation” OR “choriocarcinoma-like” OR “intermediate trophoblast” OR “epithelioid trophoblast” OR “chorionic type differentiation”) AND (“uterine neoplasms”[MeSH] OR “endometrial neoplasms”[MeSH] OR “uterine cancer” OR “endometrial cancer” OR “uterine carcinoma” OR “endometrial carcinoma” OR “uterine adenocarcinoma” OR “endometrial adenocarcinoma” OR “uterine sarcoma” OR “leiomyosarcoma” OR “malignant mixed müllerian tumor” OR “MMMT”)	PubMed	4019 results
(‘human chorionic gonadotropin’/exp OR hcg:ab,ti OR ‘human chorionic gonadotropin’:ab,ti OR ‘beta hcg’:ab,ti OR ‘β-hcg’:ab,ti OR ‘b-hcg’:ab,ti OR bhcg:ab,ti OR ‘hcg expression’:ab,ti OR ‘hcg production’:ab,ti)<br>OR<br>(‘trophoblastic differentiation’:ab,ti OR ‘trophoblastic elements’:ab,ti OR ‘trophoblastic features’:ab,ti OR ‘trophoblastic phenotype’:ab,ti OR ‘trophoblastic marker*’:ab,ti OR syncytiotrophoblast:ab,ti OR ‘choriocarcinomatous differentiation’:ab,ti OR ‘choriocarcinoma-like’:ab,ti OR ‘intermediate trophoblast’:ab,ti OR ‘epithelioid trophoblast’:ab,ti OR ‘chorionic type differentiation’:ab,ti)<br>AND<br>(‘uterus neoplasm’/exp OR ‘endometrium tumor’/exp OR ‘uterine cancer’:ab,ti OR ‘endometrial cancer’:ab,ti OR ‘uterine carcinoma’:ab,ti OR ‘endometrial carcinoma’:ab,ti OR ‘uterine adenocarcinoma’:ab,ti OR ‘endometrial adenocarcinoma’:ab,ti OR ‘uterine sarcoma’:ab,ti OR leiomyosarcoma:ab,ti OR ‘malignant mixed müllerian tumor’:ab,ti OR mmmt:ab,ti)	Embase	12469 results

(TITLE-ABS-KEY(“hcg” OR “human chorionic gonadotropin” OR “beta hcg” OR “β-hcg” OR “B-hcg” OR “BHCG” OR “hcg expression” OR “hcg production”)<br>OR<br>TITLE-ABS-KEY(“trophoblastic differentiation” OR “trophoblastic elements” OR “trophoblastic features” OR “trophoblastic phenotype” OR “trophoblastic marker*” OR “syncytiotrophoblast” OR “choriocarcinomatous differentiation” OR “choriocarcinoma-like” OR “intermediate trophoblast” OR “epithelioid trophoblast” OR “chorionic type differentiation”))<br>AND<br>(TITLE-ABS-KEY(“uterine cancer” OR “uterine neoplasm” OR “uterine carcinoma” OR “uterine adenocarcinoma” OR “uterine sarcoma” OR “leiomyosarcoma” OR “malignant mixed müllerian tumor” OR “MMMT” OR “endometrial cancer” OR “endometrial carcinoma” OR “endometrial neoplasm” OR “endometrial adenocarcinoma”))	Scopus	27 results
“trophoblastic differentiation” OR “trophoblastic features” OR “choriocarcinomatous differentiation” OR “syncytiotrophoblast” OR “intermediate trophoblast” OR “epithelioid trophoblast” OR “chorionic type differentiation” OR “β-hCG” OR “hCG expression” OR “human chorionic gonadotropin”<br>AND<br>“endometrial cancer” OR “uterine cancer” OR “uterine carcinoma” OR “endometrial carcinoma” OR “MMMT” OR “malignant mixed müllerian tumor” OR “leiomyosarcoma”	Google scholar	37 results

Supplementary Table 3: A summary of clinicopathologic features, treatment received, and patient survival/prognosis of all cases of endometrial cancers with trophoblastic differentiation reported in the literature from inception till January 2024. See Supplementary Table 3
