# Supplementary material for: Prognostic significance of trophoblastic differentiation and β-hCG secretion in somatic malignancies of uterine corpus: A systematic review with survival analysis
Source: Oncoscience. 2025 Sep 4;12:96–106. doi: 10.18632/oncoscience.625 (PMC12410977; doi:10.18632/oncoscience.625)
Supplement: Supplementary file 2 [file oncoscience-12-625-s002.docx]

**Table 1: A summary of clinicopathologic features, treatment received, and patient survival/prognosis of all cases of endometrial cancers with trophoblastic differentiation reported in the literature from inception till January 2024**

| **Author, Year of publication** | **Age/**  **Obstetric history/ Comorbidity, if any** | **Pre-operative beta HCG levels** | **Post-operative beta HCG levels** | **Treatment received** | **Histologic characteristics of the tumor** | **Tumor grade** | **pTNM stage** | **LVSI** | **Positive IHC markers in adeno-carcinomatous component** | **Positive IHC markers in the trophoblastic component** | **Time to first recurrence, if any** | **Patient prognosis (Died/survived), if died, how many months after diagnosis** |
| --- | --- | --- | --- | --- | --- | --- | --- | --- | --- | --- | --- | --- |
| Civantos et al, 1971 (4) | 87/P2L2 | 1000 IU/ 24 hour urine | - | RT | UDAC with areas of trophoblastic differentiation | HG | NM | NM | - | - | - | - |
| Tsoutsoplides GC et al, 1977 (5) | 48/P3 | positive | negative | TAH with BSO f/b RT | PDAC | HG | TxNxM, Myometrial invasion present | P | - | - | No recurrence | Alive at 36 months |
| Savage et al, 1987 (6) | 70/P1L1/  glaucoma, HT, cystocele repair, and delivery of a mentally retarded child with spina bifida | NM | NM | RT f/b TAH with BSO f/b CT with 5-FU, Adriamycin, and megestrol acetate | EC with areas of trophoblastic differentiation | G1 | NM | NM | - | β-hCG | 2 months | Died at 1 year d/t multiple metastases of CC l/t pulmonary insufficiency. |
| Pesce C et al, 1991, Case 1 (7) | 78/Nulliparous | 19,500mIU/ml | After 1^st^ CT:460mIU/ml, after 2^nd^ CT: 49 mIU/ml | Cisplatin, bleomycin, & vincristine | PDAC with trophoblastic/CC area | HG | NM | P | AE 1/AE3, EMA | β-hCG, PLAP (focal) | Advanced-stage disease at diagnosis | Died at 47 days |
| Pesce C et al, 1991, Case 2 (7) | 48/P5L5 | 3050mIU/ml | post-surgery and 2 cycles of methotrexate 17mIU/ml | surgical resection f/b 2 cycles of Mtx f/b CT for metastasis (BEP) | PDAC with trophoblastic/CC area | HG | NM | P | AE 1/AE3, EMA | β-hCG, PLAP (focal) | 2 months | NM |
| Pesce C et al, 1991, Case 3  (7) | 63/Multiparous | urinary BHCG-100000 IU/24 hours | - | TAH+BSO | PDAC with trophoblastic/CC area | HG | NM | P | AE 1/AE3, EMA | β-hCG, PLAP (focal) | 12 months | Died after 2 months of recurrence |
| Kalir et al, 1995  (8) | 83/Nulliparous/  Diverticulitis, osteoporosis,  multiple fractures | >100,000 IU/ml | 19,200 (D-5) and 24000 (D-10). | TAH with BSO f/b CT with Cisplatin and Etoposide | EC with focal trophoblastic differentiation | G2 | NM | NM | ß-hCG (weak, focal) | ß-hCG | 1 month | Not mentioned |
| Molina et al, 1996  (9) | 85/Multiparous/ HT, unilateral nephrectomy due to obstructive cause | NA | NA | Surgical Staging | ESC without trophoblastic differentiation | HG | pT3aNx | P | CAM 5.2, EMA | β-hCG, CAM 5.2, EMA (weak, focal) | Died at 14 days, no recurrence. | DIED at 14 days, Unexplained |
| Bradley et al, 1998 (10) | 68/P4L4/  HT, DM, COPD | 95 mIU/ml | Undetectable after surgery and CT | Surgical Staging f/b CT with C/P (6 cycles), megestrol acetate | EC with areas of squamous (minor), clear cell (20%), and trophoblastic differentiation | G3 | pT1bN1M | P | - | ß-hCG | No Recurrence | Alive and healthy at 16 months |
| Black K et al, 1998 (11) | 88/P1L1/  AF, COPD | not measured | 851 IU/L .  (PO-21) - 6 IU/L. | Surgical Staging f/b pelvic megavoltage RT 50.4 Gy in 28 fractions. | CCAD with areas of trophoblastic differentiation | HG | pT1aNx | NM | - | - | None reported | Died at 18 months, d/t septic shock and ARF following UTI |
| Nguyen CP et al, 2000 (12) | 34/Nulligravida/  morbid obesity, HT, and chronic anemia | 32,000 mIU/mL | After NACT–9600mIU/ml  PO - 4800 mIU/mL,  After 6 cycles of EMACO-637,  Increased again to 6000, given 2 cycles of C/P - 5000. | 2 cycles of BEP (NACT), Surgical Staging, After surgery EMACO, then changed to C/P, due to poor response. | uterine MMMT with trophoblastic/CC area | HG | pTxN2 | P | p53, MSA | p53, β-hCG | 5 months | Died at 6 months d/t to haemorrhage from cerebral metastasis. |
| Khuu HM et al, 2000  (13) | 71/Nulliparous/  Mild mental retardation | not measured | 283 (PO 14), 32 (PO 27), and 7 mIU/mL (PO 42). Thereafter,  < 5 mIU/mL | Surgical Staging | Carcinosarcoma with trophoblastic differentiation | HG | pTxN0 | P | vimentin | β-hCG, hPL | No recurrence | Alive and Healthy at 9 months |
| Yasuda M et al, 2004  (14) | 31/NA | pre-op CA125:120 U/mL | not measured | Surgical Staging | EC with areas of yolk sac-like and trophoblastic differentiation | G3 | pTxN1 | NM | AFP (focal), PLAP (focal) | β-hCG | 7 months | Died at 7 months, with extensive disease |
| Grenache et al, 2004  (15) | 24/Nulliparous | Not available | Week 0- 56, Week 4- 45, week 6- 60, week 10-94, week 15-201 | RT | EC without trophoblastic differentiation | G2 | NM | NM | β-hCG | β-hCG | NM | NM |
| Liang et al, 2006  (16) | 49/P4L4/  Fibroid Uterus | NM | NM | Surgical Staging | LMS with  No definitive trophoblastic/CC foci | HG | NM | P | vimentin, SMA, HHF-35, desmin, ß-hCG | - | 15 days | Died at 1 month due to multiple metastases, l/t bowel obstruction |
| Horn LC et al, 2006, 2008  (17, 18) | 61/P3L3 | Not available | 225,000 IU/L | TAH with BSO. CT with single agent Mtx. After 3 cycles, due to an increase in hCG, switched to the EMACO X 5 courses. | ESC with areas of trophoblastic (35%) differentiation | HG | T2bN0 | A | p53 | p53 | 2 months | Died at 2 months, Metastatic disease and immunodeficiency due to chemotherapy, leading to pneumonia |
| Akbulut M et al, 2008 (19) | 42/NA | not measured | 1, 74 mIU/mL | Surgical Staging | EC with areas of trophoblastic differentiation | NM | pT1cN0M0 | P | CK, β-hCG, ER (focal), PR(focal), HER2 (score 3), p53 | CK, β-hCG | No recurrence | alive at 6 months follow-up |
| Yamada et al, 2009 (20) | 58/P1L1 | Not available | β-hCG - 2.3 mIU/mL (PO 53) and 0.1 mIU/mL (PO 83). | Surgical Staging f/b 5 courses of the CTP. Recurrence at vaginal cuff- 6 courses of EMACO given | EC with areas of trophoblastic (50%) differentiation | G1 | pT1aN0 | P | CA125, vimentin, AE1/AE3 | β-hCG, AE1/AE3 | 9 months | Alive at 3 years |
| Wakahashi et al, 2011 (21) | 85/P5L5 | 8.0 ng/ml | Twenty weeks later, serum hCG-β subunit was elevated to 55 ng/ml. | Surgical Staging. CT not given, considering the patient's age | EC with areas of squamous and trophoblastic differentiation | NM | p T3aNx | P | p53 | β-hCG, p53 | 5 months | alive at 5 months with complications. Bowel obstruction needing a diverting colostomy. |
| Olson MT et al, 2011 (22) | 68/  P 4-0-2-4/  Intraductal high-grade papillary carcinoma of the breast | NM | NM | TAH with BSO & modified radical mastectomy. | EC with areas of choriocarcinoma and epithelioid trophoblastic tumor (ETT) | G3 | NM | P | CK 20 (focal) | HSD3B1, β-hCG, and CD10 in both CC and ETT. p63 in ETT | NM | NM |
| Seki et al, 2013 (23) | 54/P2L2 | Not done | Post-operative 1632 mIU/ml.  After primary CT-8318, which decreased to 291 after 2nd-line CT with MEA. | Surgical Staging f/b CT with doxorubicin and Cisplatin, every 21 days.  Due to poor response, changed to MEA, but the response was still not adequate | EC with areas of trophoblastic (5%) differentiation | NM | pT1bNx | NM | - | β-hCG, hPL, inhibin-α | Multiple lung nodules were present at diagnosis | Died at 1 year d/t brain metastasis. |
| Mingliang Ji et al, 2013 (24) | 28/Nulligravida/  Epilepsy, hypothyroidism | 518.9IU/ml | 13.7IU/L | Surgical Staging f/b CT with Cb/P every 21 days. 2 cycles of EMACO, salvage chemotherapy with two cycles of floxuridine, dactinomycin, etoposide, and vincristine | EC with yolk sac tumor-like areas | G2 | TxN1M1 | NM | AE1/AE3,EMA, CA125, CK7, p53(focal) | The yolk sac tumor component was AFP | lost to F/U | lost to follow up |
| Mitsuaki Ishida et al, 2013 (25) | 59/NA  Scleroderma, DM | not done | not done | Surgical Staging | EC with areas of trophoblastic (20%) differentiation | G3 | p T3a N1 | P | AE1/AE3, CA125 | β-hCG | NM | - |
| Carta et al, 2014 (26) | 50/Nulliparous | Not done | Negative | TAH with BSO | EC with focal trophoblastic differentiation | G1 | pT1aNxM | NM | CK 7, AE1/AE3 | CK7, AE1/AE3, β-hCG, HER2 | None | Alive and healthy at 24 months |
| Lee R et al, 2015 (27) | 64/P1L1/  HT, DM | 7431 mIU/ml | 14,361 (D-10)  After RT- 11,915, After EMACO (1 Cycle)– 2701,  Before 2^nd^cycle - 42,  Normal after EMACO | Surgical Staging f/b CT with EMACO and RT with 30 Gy to pelvis over 5 days to the vaginal cuff to reduce bleeding. | ESC with clear cell areas and trophoblastic differentiation | HG | pT1bN1 | P | p53 | - | 1.5 months | Alive and healthy at 20 months |
| Cai H et al, 2018 (28) | 33/Nulliparous/  schizophrenia | 238,418.35 U/L | 43637.42 U/L after 2 days and 2055 U/L after 28 day | Surgical Staging f/b BEP | EC with areas of trophoblastic differentiation | G3 | pT1bNxM1 | NM | CK7, EMA, vimentin | β-hCG, hPL, PLAP | 3 weeks | died at 5 months, d/t extensive disease |
| Yadav S et al, 2018 (29) | 68/NA | 4.2 ng/ml. | not done | Surgical Staging | ESC with areas of trophoblastic (90%) differentiation | HG | TxNxM1, Myometrial invasion present | NM | EMA, AE1/AE3, p53 | EMA, AE1/AE3, β-hCG, hPL | No recurrence | Alive and Healthy at 4 months |
| Rawish KR et al, 2018, case 1 (30) | 61/Nulliparous | not measured | PO- 5 months - 81,900mIU/mL | Surgical Staging f/b EMACO f/b EMA-EP | EC with foci of mucinous, squamous, and trophoblastic differentiation | G2 | pT1a N0 M0 | A | p63, p53, PAX8, e-cadherin, AE1/AE3,  ER, PR | GATA-3, SALL4, p63 , p53,e-cadherin, AE1/AE3, β-hCG, PLAP, hPL, p40 | 5 months | Died at 17 months, due to metastasis |
| Rawish KR et al, 2018, case 2 (30) | 72/P4L3 | not measured | PO- 15: 41,848mIU/mL | Surgical Staging, EMA-CO f/b C/P f/b RT | EC with areas of trophoblastic differentiation | G3 | pT3a N0 M0 | P | p63, p53, PAX8, e-cadherin, AE1/AE3,  ER, PR, SALL4 (focal, weak), PLAP (focal, weak) | GATA-3, SALL4, p63 , p53,e-cadherin, AE1/AE3, β-hCG, PLAP, hPL, p40 | 2.5 months | Died at 7 months, due to metastasis |
| Rawish KR et al, 2018, case 3 (30) | 77/P5L4 | 68 mIU/mL | (PO-30) 8.8 mIU/mL | Surgical Staging, EMA-EP (VP-16, MTX and actinomycin D, then VP-16 and cisplatin) | EC with areas of trophoblastic differentiation | G1 | pT3a N2 M0 | P | PAX8, AE1/AE3 | GATA-3, SALL4, p63, AE1/AE3, β-hCG, PLAP | 10 months | died at 11 months, due to metastasis |
| Rawish KR et al, 2018, case 4  (30) | 62/P1L1 | not measured | (PO-7): 6840 IU/L | Surgical Staging f/b EMA-CO f/b TP-TE (Taxol and cisplatin then Taxol and VP-16) | EC with areas of trophoblastic differentiation | G3 | pT3a N1 M0 | P | p53, PAX8, e-cadherin, AE1/AE3  ER, PR | GATA-3, SALL4, p63, p53, PAX8,e-cadherin , AE1/AE3, β-hCG, PLAP , HPL , P40 | 7 months | died at 16 months, due to metastasis |
| Mathew S et al, 2019 (31) | 50/NA | 628 IU/ml | NM | TAH with BSO f/b CT, but details not available | PDAC with areas of trophoblastic differentiation | HG | NM | NM | - | - | Developed lung mets, but the time is not mentioned. | Died, due to multiple lung metastases, but the time is not mentioned |
| Krishnan et al, 2020 (32) | 33/P0A4/  Fibroid Uterus | At presentation, 49.7 U/L, peak at 90.1 U/L before surgery. | After surgery, 0.7 U/L and 1.1 U/L at 3 months postop. | Surgical Staging, Planned CT with Gemcitabine and Docetaxel | pleomorphic uterine sarcoma with  No definitive trophoblastic/CC foci | HG | NM | NM | ß-hCG (focal) | - | 6 months | Alive at 6 months, with multiple lung metastases |
| Syeda et al, 2020 (33) | 55/NA | 9421 mIU/ml | After surgery -84 (D10), 25 (D15) | Surgical Staging f/b CT with Docetaxel, nedaplatin every 21 days, 4 cycles | EC with areas of trophoblastic differentiation | G3 | pT1aNx | P | Pan CK, CK8/18, CD 10 | β-hCG | None | alive and healthy at 48 months. Follow-up was done as in CC, with Beta hCG every 3 months for 2 years. |
| Tsakos et al, 2022 (34) | 54/Nulliparous/  DM | 383.3 IU/L | (PO-21) – Normal  (2.2 IU/L). | Surgical Staging | LMS,  No definitive trophoblastic/CC foci | HG | NM | NM | SMA, h-caldesmon, desmin | ΑΕ1/ ΑΕ3, β-hCG | None reported | Died at 6 months, due to pulmonary embolism. |
| Meiping Li et al, 2022 (35) | 68/P2L2/ | 6374IU/L | Post-surgery and CT -24,867IU/L | Surgical Staging f/b CT with 6 cycle Cb/P, f/b 4 courses of  Act-D and 5-Fu | ESC with trophoblastic (70%) differentiation | HG | pT4N0M | P | p53, IMP3, Pax8 | SALL4 , GATA3 , ß-hCG, PD-L1 | 13 months | Died at 13 months |
| Bai L et al, 2023 (36) | 38/Nulligravida | 36,774.5IU/ml | After 5 cycles of EMACO 37.3IU/ml.  After surgery Normal | NACT with EMACO - 5 cycles f/b TLH+BSO f/b  4 EMACO post op and 33 cycles of CT/RT - 51.7 Gy | EC with focal trophoblastic differentiation | NM | p T3a N1M1 | P | ER,PR,p16 | - | Metastasis present at the time of diagnosis | Alive and healthy at 40 months |
| Xie Y et al, 2022 (37) | 39/Nulliparous | 52,247.8 mIU/mL | After each cycle -1^st^ - 52,247.8,  2^nd^ -36,305.2,  3^rd^- 4,349.5,  4^th^ -248.1,  5^th^ - 35.3,  (PO-21) –  <2 mIU/ml & normal ever since. | 5 course of NACT (2 courses of the Mtx + VP16, 3 courses of the EMACO. f/b TAH with BSO f/b Postoperatively, EMACO X 4 cycles and 33 sessions of RT | EC with trophoblastic (85%) differentiation | G1 | pT1aNx | P | ER, PR | β-hCG, PLAP (focal) | No recurrence | Alive and Healthy at 37 months |
| Zhang Z et al, 2023 (38) | 60/P2L1 | 192193 mIU/ml | After NACT- 497 mIU/mL.  After three cycles of post-operative CT-4.3 mIU/mL.  9 weeks after stopping CT - 73 mIU/mL. | 3 cycles NACT with low-dose etoposide-cisplatin f/b TLH with BSO, CT (PO-3) full-dose C/P alternating with paclitaxel/etoposide and 3 cycles of consolidation CT, the 3rd of which was changed to 3-weekly paclitaxel/carbo- platin d/t myelosuppression.  RT (28 EBRT and 2 courses of brachytherapy. | CCAD with areas of trophoblastic differentiation | HG | NM | NM | HNF1B, PAX-8, p53 | β-hCG | 2 months | Alive and Healthy at 42 months |
| Alzibdeh et al, 2023 (39) | 44/NA | 0.11 mIU/ml | 8.43 mIU/ml | TAH with BSO f/b palliative CT/RT | LMS, no definitive trophoblastic/CC foci | HG | pT1bNxM | NM | desmin, SMA, h-caldesmon, CD10 (focal, weak) | - | 15 months | died d/t MODS |

A: absent; AFP: Alpha-fetoprotein; BEP: Bleomycin, Etoposide and Cisplatin; CC: chorio-carcinomatous; CK: cytokeratin; CCAD: Clear cell adenocarcinoma, NOS of endometrium; CTP: Carboplatin, therarubicin, and cyclophosphamide; COPD- Chronic Obstructive pulmonary disease; C/P: Cisplatin plus Paclitaxel; Cb/P: Carboplatin plus paclitaxel; CT: Chemotherapy; DM- Diabetes mellitus; EMA: Epithelial membrane antigen; ESC:Serous carcinoma of endometrium; EC: Endometrioid carcinoma, NOS of endometrium; f/b: followed by; , 5-FU:5-fluorouracil; HG: High grade; hPL: human placental lactogen; HT- Hypertension; IHC: Immunohistochemical; MEA: methotrexate, etoposide, and actinomycin D; Mtx: methotrexate; MMMT: Malignant Mixed Mullerian tumor; MODS- Multiorgan dysfunction syndrome, MSA: muscle-specific actin; NA: not available; NACT: Neoadjuvant Chemotherapy; LMS: Leiomyosarcoma; LVSI: Lympho-vascular space invasion; NM: not mentioned in the concerned article; PDAC: Poorly differentiated adenocarcinoma of endometrium; P: Present; PLAP: Placental alkaline phosphatase; RT: Radiotherapy; SMA: Smooth muscle actin; TAH with BSO:Total abdominal hysterectomy with bilateral salpingo-oophorectomy; UDAC: undifferentiated adenocarcinoma of endometrium
